# The Extracts of Dendrobium Alleviate Dry Eye Disease in Rat Model by Regulating Aquaporin Expression and MAPKs/NF-κB Signalling

**DOI:** 10.3390/ijms231911195

**Published:** 2022-09-23

**Authors:** Jiawei Ling, Chung-Lap Chan, Chi-Yan Ho, Xun Gao, Sin-Man Tsang, Ping-Chung Leung, Jiang-Miao Hu, Chun-Kwok Wong

**Affiliations:** 1 State Key Laboratory of Research on Bioactivities, Institute of Chinese Medicine, Clinical Applications of Medicinal Plants, The Chinese University of Hong Kong, Hong Kong, China; 2 Center of Clinical Laboratory Medicine, Zhongda Hospital, Southeast University, Nanjing 211189, China; 3 Department of Chemical Pathology, Prince of Wales Hospital, The Chinese University of Hong Kong, Hong Kong, China; 4 State Key Laboratory of Phytochemistry and Plant Resources in West China, Kunming Institute of Botany, Chinese Academy of Sciences, Kunming 650204, China; 5 Li Dak Sum Yip Yio Chin R & D Centre for Chinese Medicine, The Chinese University of Hong Kong, Hong Kong, China

**Keywords:** dry eye disease, Dendrobium, aquaporin, scopolamine, human cornea keratocytes

## Abstract

Dry eye is one of the most common ocular surface diseases caused by tear film instability and ocular surface damage due to an abnormal quality or quantity of tears. Inflammatory factors can initiate relevant transduction signalling pathways and trigger the inflammatory cascade response, resulting in ocular surface inflammation. It has been shown that the active ingredients in Dendrobium, such as polysaccharides, alkaloids and phenols, have anti-inflammatory, anti-tumour and immunity-boosting effects, and Dendrobium officinale extract can improve glandular secretion function, increase salivary secretion and increase the expression level of water channel protein in salivary glands in patients with dry eye syndromes. We investigated the in vitro cytoprotective effect of Dendrobium extracts in sodium chloride induced hyperosmotic conditions in human cornea keratocytes (HKs). Results showed that Dendrobium officinale Kimura et Migo water extract (DOW) and Dendrobium loddigesii Rolfe water extract (DLW) could upregulate the expression of aquaporins (AQP)5 protein, thus exerting a repairing effect by promoting cell migration. Furthermore, oral administration of DOW and DLW enhanced tear production in rats and exerted a protective effect on ocular surface damage. DOW and DLW could upregulate the expression of AQP5 and mucin (muc)5ac proteins in the lacrimal gland and reduce the inflammatory response. DOW and DLW inhibited the activation of the corresponding mitogen-activated protein kinases (MAPK) and NF-KB pathway, thereby playing a role in improving dry eye symptoms. This study provides a new perspective on dry eye treatment, and DOW and DLW may be potential therapeutic agents for dry eye.

## 1. Introduction

Dry eye is an umbrella term for dry eye syndromes (DES) and dry eye disease (DED). It is defined as a multifactorial disorder of tear film and ocular surface dysfunction with ocular discomfort, visual disturbances, tear film instability and increased tear osmolarity and inflammation of the ocular surface [1]. The DEWS II (Dry Eye Workshop II) 2017 conference summary report included sensory nerve abnormalities in the definition for the first time [2]. Compared with the 2007 DEW I definition, it retained the core mechanism of ocular surface inflammation and emphasized the vital role of damage to the corneal nerve in the pathogenesis of dry eye. Currently, the pathogenesis of dry eye has not fully defined, but clinical and experimental studies have shown that dry eye is a chronic inflammatory disease [3,4]. In addition, epidemiological studies have shown that the incidence of dry eye increases with age [5]. Aging is inextricably linked to oxidative stress [6], and environmental factors are often associated with dry eye, including atmospheric pollution, ultraviolet radiation, ozone, and long-term use of preservative-containing eye drops [7]. All of the above can cause oxidative stress and inflammation of the ocular surface. However, the current treatment for dry eye varies widely in clinical efficacy, and the results are still unsatisfactory.

It has been shown that tumour growth, inflammation, immune response, cell growth and differentiation, infectious shock, viral infection and immune system dysplasia are all associated with an imbalance in inflammatory transcription factor NF-κB regulation [8,9]. NF-κB has recently been reported to play an important role in the development of ocular diseases, such as cataracts, glaucoma and retinopathy, and the development of related ocular diseases can be suppressed by inhibiting NF-κB activity [10]. Lee et al. [11] used ozone to induce a dry eye model and found that ozone led to corneal epithelial disruption and a decrease in the number of mucin-secreting cells, induced inflammatory cytokine production without altering tear volume and caused increased NF-κB p65 nuclear translocation, IκBα protein hydrolysis and phospho (p)-IκBα expression. Li et al. [12] used a novel dry eye mouse model induced by air pollutant particulate matter 10 (PM10), which disrupted tear film function and ocular surface tissue structure and significantly increased corneal TNF-α, p65 and phospho-p65 expression. In summary, these results suggest that targeted inhibition of NF-κB activation may have a therapeutic effect on dry eye disease. Intracellular p38 mitogen-activated protein kinase (MAPK) can be upregulated by a variety of stimuli, such as extracellular inflammatory factors, stress response and reactive oxygen species activation, and is involved in the apoptosis regulation process [13,14]. Many studies have confirmed [15] that the expression of most cellular inflammatory factors is significantly enhanced in the pathogenesis of dry eye. The expression and release of these inflammatory factors are also closely related to the activation of extracellular signal-regulated kinase (ERK) and p38 MAPK signalling pathways. Moreover, by interfering with ERK and p38 signalling pathways, the transduction of inflammatory signals toward the apoptosis in the dry eye process can be inhibited [16,17].

The genus Dendrobium is the second largest genus of orchids, with 76 species in China, and nearly 40 species can be used medicinally [18]. The medicinal use of Dendrobium, which is a traditional and valuable Chinese medicine in China, was first published in *Shennong Ben Cao Jing*, with the effects of benefitting the stomach; nourishing Yin and clearing heat; mainly treating heat illnesses and injury to the body; and relieving stomach pain and vomiting, lung and cough and weakness of the waist and knees [19]. Dendrobium mainly contains chemical components including polysaccharides, flavonoids, bibenzyls, phenanthrenes, alkaloids, amino acids, etc. [20]. Modern research shows that it has pharmacological effects, such as enhancing body immunity, promoting digestive secretion, inhibiting platelet agglutination, lowering blood lipids, lowering blood sugar and providing anti-tumour, anti-oxidation and anti-aging activities [21]. Dendrobium officinale extract can improve glandular secretion function and increase salivary secretion and the expression level of water channel protein in salivary glands in patients with dry eye syndromes [22]. The incidence of dry eye is high, but there are not many treatment modalities that can be widely used effectively or that are recognized by the public in modern medicine, except for artificial tear replacement therapy. Therefore, it is feasible to select Dendrobium, a classical medicinal and food source, to study the alternative treatment of dry eye disease. The purpose of this study is to investigate the protective and aquaporin-modulating effects of selected Dendrobium on chemically induced experimental dry eye disease and to investigate the underlying mechanisms.

## 2. Results

### 2.1. Dendrobium Officinale Kimura et Migo Water Extract (DOW) and Dendrobium Loddigesii Rolfe Water Extract (DLW) Promote Cell Migration on the Hyperosmotic HKs Model

**Figure 1:**
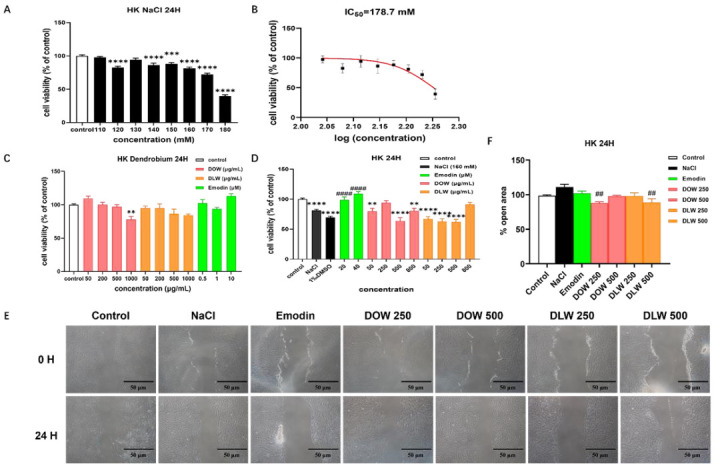
DOW and DLW promote cell migration on the hyperosmotic HKs model. (**A**) The effect of different concentrations of NaCl on the cell viability of HKs by MTT assay. *** *p* < 0.001 and **** *p* < 0.0001 compared to control individually, *n* = 3. (**B**) Calculation of IC50 values for NaCl on HKs by Graphpad 9.0. (**C**) Cytotoxicity of Dendrobium extracts on HKs by MTT assay. ** *p* < 0.01 compared to control individually, *n* = 3. (**D**) Cytotoxicity of Dendrobium extracts on the hyperosmotic HKs model by MTT assay. ** *p* < 0.01 and **** *p* < 0.0001 compared to control individually, #### *p* < 0.0001 compared to 1% DMSO group individually, *n* = 3. (**E**) Representative photos of the scratch assay of DOW and DLW on HKs. (**F**) Statistical analysis of open area of each group using TScratch software. ## *p* < 0.01 compared to NaCl group individually, *n* = 3.

We first examined the effect of different concentrations of NaCl on the cell viability of HKs (Figure 1A). Emodin was used as a positive control, as it has been reported to exert effect to modulate the aquaporins (AQP)3 protein expression [23]. As shown in Figure 1B, we calculated the IC_50_ value of NaCl on HKs to be 178.7 mM. DLW exhibited no significant cytotoxicity to HKs at the selected concentrations (all *p* > 0.05). DOW was significantly toxic to cells at a concentration of 1000 μg/mL (*p* < 0.01). We then investigated the effect of different concentrations of Dendrobium extracts on the hyperosmotic HKs model. As shown in Figure 1C,D, neither DOW nor DLW exerted a protective effect on hyperosmolarity-induced cell death, at different doses, except DOW (250 μg/mL) and DLW (800 μg/mL). In subsequent experiments, we chose a 160 mM NaCl concentration to induce a hyperosmotic model in HKs. DOW and DLW (250 and 500 μg/mL) had no effect on the proliferation of HKs and did not exert any protective effect on cells under hyperosmotic conditions (Figure 1C,D). In our experimental results (Figure 1E,F), DOW and DLW had no significant effect on cell proliferation and did not affect its repair-promoting effect, but both could promote the migration of HKs to exert the repair function.

### 2.2. DOW and DLW Modulate the Expression of AQPs and Downregulate MAPKs and NF-κB Signal Transduction on the Hyperosmotic HKs Model

**Figure 2:**
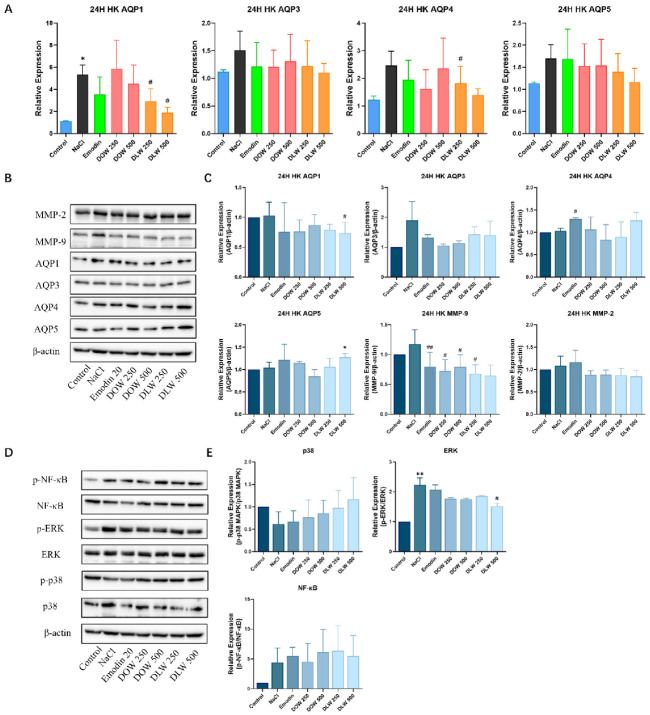
DOW and DLW modulate the expression of AQPs and downregulate MAPKs and NF-κB signal transduction on the hyperosmotic HKs model. (**A**) qRT-PCR for gene expression of AQPs of Dendrobium extracts on the hyperosmotic HKs model. * *p* < 0.05 compared to control individually, # *p* < 0.05 compared to NaCl individually, *n* = 3. (**B**) Representative bands selected denote the protein expression of AQPs and MMPs of the effect of Dendrobium extracts on the hyperosmotic HKs model by Western blot in each group (*n* = 3). (**C**) Quantitative statistics of protein expression in Figure 2B. The grey value was obtained by Image J software processing. * *p* < 0.05 compared to control individually, # *p* < 0.05 and ## *p* < 0.01 compared to NaCl treatment group, *n* = 3. (**D**) Representative bands selected denote the protein expression of MAPKs pathway and NF-κB pathway of Dendrobium extracts on the hyperosmotic HKs model by Western blot in each group (*n* = 3). (**E**) Quantitative statistics of protein expression in Figure 2D. The grey value was obtained by Image J software processing. ** *p* < 0.01 compared to control individually; # *p* < 0.05 compared to NaCl individually, *n* = 3.

We investigated the effect of different concentrations of Dendrobium extracts on the gene expression of AQPs in the hyperosmotic HKs model. As shown in Figure 2A, NaCl induced hyperosmolarity increased the gene expression of AQP1 (*p* < 0.05), AQP3, AQP4 and AQP5. Compared with the NaCl group, DLW could significantly reduce the gene expression of AQP1 (*p* < 0.05) in a dose-dependent manner, and DLW had a trend of reducing the gene expression of AQP1, AQP4 (*p* < 0.05) and AQP5 to a normal level in a dose-dependent manner. As shown in Figure 2C, compared with the control group, NaCl induced hyperosmolarity has a tendency to increase the protein expression of AQP3 and MMP-9. Compared with the NaCl group, DLW (500 μg/mL) could significantly decrease the protein expression of AQP1 (*p* < 0.01). Compared with the control group, DLW at 500 μg/mL could significantly increase the protein expression of AQP5 (*p* < 0.05). Compared with the NaCl group, both DOW and DLW could reduce the expression of MMP-9 (*p* < 0.05). Moreover, compared with the control group (Figure 2E), the NaCl group could increase the expression of phospho-NF-κB, especially phospho-ERK (*p* < 0.01). Compared with the model group, DLW (500 μg/mL) could decrease the protein expression of phospho-ERK in a dose-dependent manner (*p* < 0.05).

### 2.3. DOW and DLW Increase Tear Secretion and Inhibit Loss of Goblet Cells in Scopolamine (SCOP)-Induced DED Rats

**Figure 3:**
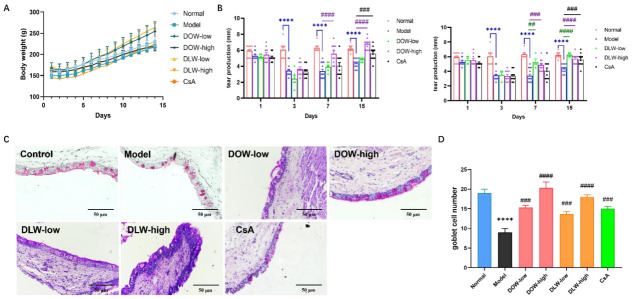
DOW and DLW enhanced tear secretion and inhibited the loss of goblet cells in SCOP-induced DED rats. (**A**) The body weight of rats in all groups maintained a stable increase that was not affected by SCOP injection or treatment for 14 days. Data were expressed as mean ± SEM from five rats in each group. (**B**) Changes in tear production. Results were expressed as mean ± SEM from five rats in each group, **** *p* < 0.0001 compared to control group; ## *p* < 0.01, ### *p* < 0.001 and #### *p* < 0.0001 compared to model group individually. (**C**) Effects of DOW and DLW on morphological changes of conjunctival goblet cells in rats by using PAS staining in each group. Original magnification × 200, scale bar = 50 μm. (**D**) Conjunctival goblet cell count of rats in each group. Data were expressed as mean ± SEM from five rats in each group. **** *p* < 0.0001 compared to control group; ### *p* < 0.001 and #### *p* < 0.0001 compared to model group individually.

As shown in Figure 3A, there was no statistically significant difference in body weight between the groups. The bodyweight of rats in each group increased steadily with time. The experimental results show that the method of subcutaneous injection of SCOP to cause dry eye in rats did not affect the body weight of the rats. At the same time, DOW and DLW did not have a systemic toxic effect on rats. Meanwhile, according to our observation during the experiment period, there is no abnormal behaviour or loss of welfare in all rats. Additionally, Dendrobium itself is a traditional Chinese medicine, which can be used both as food and medicine, and it is widely used in daily diet. The experimental doses we adopted were converted from the human doses described in the *Chinese Pharmacopoeia*, which were all within the safe dosage range. The amount of tear production mainly reflects the function of the lacrimal gland, which can help to diagnose tear-deficient dry eye in clinical practice. The amount of tear production is an important indicator to judge whether a dry eye animal model is successfully induced. On the third day, the tear production of all SCOP-injection rats was significantly lower than that of the control group (*p* < 0.001), which proved that the dry eye model was successfully established by subcutaneous injection with scopolamine (Figure 3B). On the 7th day, the model group still maintained a low level of tear production, which was significantly different from the control group (*p* < 0.001). At this time, the treatment group had been administered for 4 days, and the DOW and DLW group had a tendency to increase tear production, which was statistically significant (*p* < 0.001) compared with the model group. On the 15th day, the model group still maintained a low level of tear production, while the DOW-high group completely recovered tear production to the level of the control group (*p* < 0.0001). The CsA group also significantly improved the tear production in rats (*p* < 0.001). Conjunctival goblet cells play an essential role in the stability of the tear film. It can be observed in Figure 3C that there are goblet cells in the fornix of the conjunctiva of the rats in the control group, with clear layers, cylindrical appearance and orderly arrangement, and goblet cells of uniform size are distributed among them. In the model group, the epithelial cells in the conjunctiva of the rats showed the disordered arrangement and hyperplasia. The data analysis chart of conjunctiva goblet cell numbers from Figure 3D demonstrates that the goblet cells in the model group decreased compared with the control group (*p* < 0.0001). Comparing with the model group, both the DOW and DLW group (*p* < 0.0001) and the CsA group (*p* < 0.001) had different degrees of increase in the number of goblet cells.

### 2.4. DOW and DLW Maintain the Ocular Surface Barrier in SCOP-Induced DED Rats

**Figure 4:**
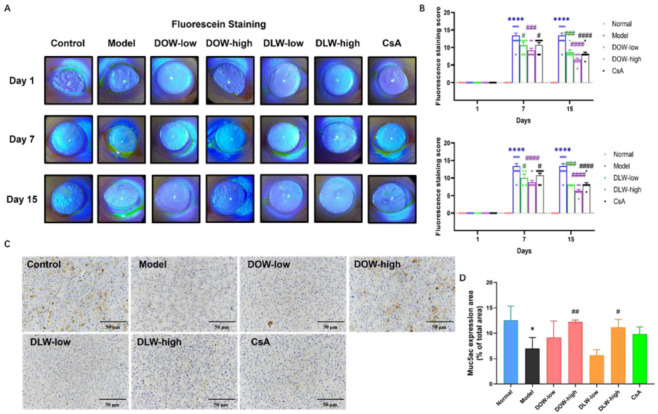
DOW and DLW maintain the ocular surface barrier in SCOP-induced DED rats. (**A**) Fluorescein staining with 1% fluorescein sodium; photos were captured under a blue filter of the slit lamp. (**B**) Corneal opacity scores were assessed on days 1, 7 and 15. Data were expressed as mean ± SEM from five rats in each group, **** *p* < 0.0001 compared to control group; # *p* < 0.05, ## *p* < 0.01, ### *p* < 0.001 and #### *p* < 0.0001 compared to model group individually. (**C**) Morphology of immunohistochemistry (IHC) staining of muc5ac expression in the lacrimal gland of each group. Original magnification × 200, scale bar = 50 μm. (**D**) Muc5ac expression was quantified as percentage of total area. Data were expressed as mean ± SEM from five rats in each group. * *p* < 0.05 compared to control group; # *p* < 0.05 and ## *p* < 0.01 compared to model group individually.

Fluorescein sodium staining score is one of the common ophthalmic clinical examination methods, which is mainly used to check whether there is a defect in the corneal epithelium and to detect the degree of damage. In this experiment, the staining of sodium fluorescein on the corneal epithelium of rats in each group was observed under the blue light of a slit lamp. Figure 4A,B shows the results of the fluorescein sodium score of the rats in each group and the results of the visual staining pictures. On the 7th day, the fluorescein sodium staining score of the model group was significantly and much higher than that of the control group (*p* < 0.0001), which further proves the successful induction of the dry eye model. Comparing the DOW and DLW group with the model group, the fluorescein sodium staining scores were significantly decreased (*p* < 0.01), and the CsA group could also significantly reduce the fluorescein sodium staining scores compared with the model group (*p* < 0.05). On the 15th day, compared with the model group, the fluorescein sodium staining scores in both the treatment groups significantly decreased (*p* < 0.0001).

Mucin (Muc)5ac is the most important type of ocular surface secreted mucin, and its normal expression can maintain the stability of the tear film and reduce the damage of ocular surface epithelial cells. Figure 4C,D show that the IHC staining degree of muc5ac in the model group was significantly reduced compared with the control group. Compared with the model group, the DOW-high and DLW-high group could improve the decreased secretion of muc5ac in the lacrimal gland acinar cells caused by dry eye (*p* < 0.05), but no significant improvement was found in the CsA group.

### 2.5. DOW and DLW Alleviate Ocular Disruption and Maintain Normal Eye Structure in SCOP-Induced DED Rats

**Figure 5:**
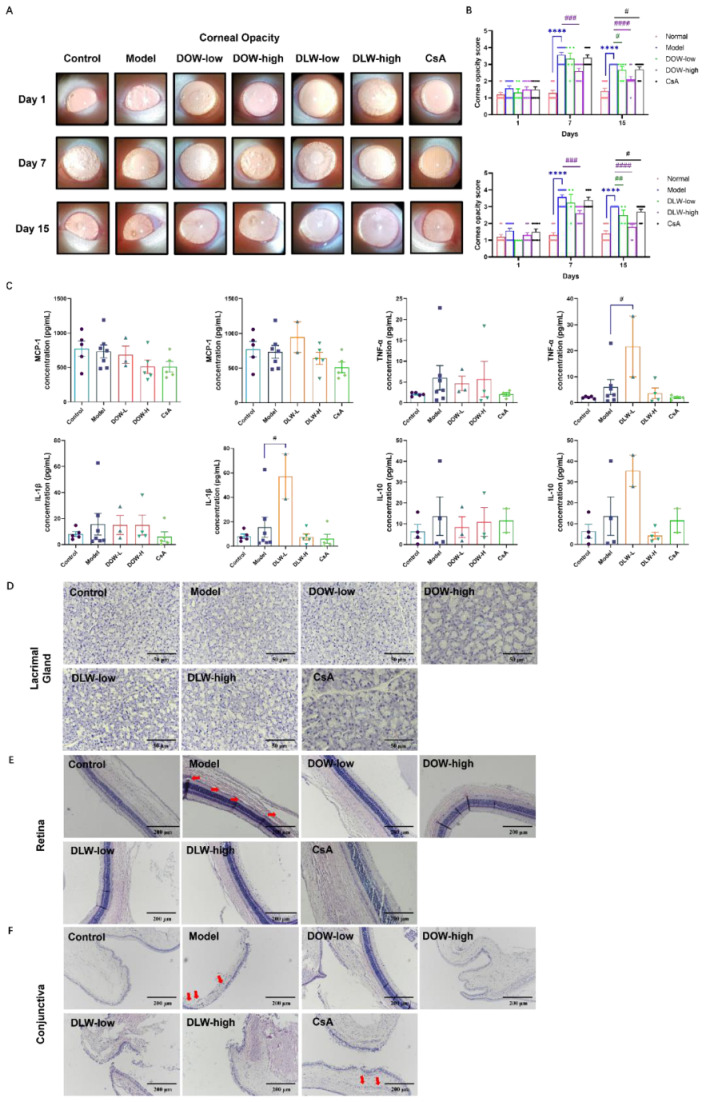
DOW and DLW alleviate ocular disruption and maintain normal eye structure in SCOP-induced DED rats. (**A**) Corneal opacity and neovascularization, photos were captured under a white filter of the slit lamp. (**B**) Corneal opacity scores were assessed on days 1, 7 and 15. Data were expressed as mean ± SEM from five rats in each group. **** *p* < 0.0001 compared to control group; ## *p* < 0.01, ### *p* < 0.001 and #### *p* < 0.0001 compared to model group individually. (**C**) Cytokine concentrations in serum of rats were measured by MILLIPLEX. Data were expressed as mean ± SEM from five rats in each group. # *p* < 0.05 compared to model group individually. (**D**) Effects of DOW and DLW on morphological changes in lacrimal gland by HE staining. Original magnification × 200, scale bar = 50 μm. (**E**) Effects of DOW and DLW on morphological changes in retina by HE staining. Original magnification × 100, scale bar = 200 μm; red arrow indicates the vacuolar changes in retina layers. (**F**) Effects of DOW and DLW on morphological changes in conjunctiva by HE staining. Original magnification (**D**–**F**) × 100, scale bar = 200 μm; red arrow indicates the hyperplasia in conjunctiva.

The degree of corneal opacity in the model group continued to aggravate after modelling. On the 7th day, degree scores of the corneal opacity in the model group were significantly higher than those of the control group (*p* < 0.0001). Compared with the model group, the DOW-high and DLW-high group could significantly improve the corneal opacity and inflammation-stimulated angiogenesis caused by SCOP (*p* < 0.001). On the 15th day, the corneal opacity of the model group was also improved, and the corresponding score decreased. Compared with the model group, all DOW and DLW treatment group could further restore the corneal opacity caused by SCOP, and the difference in related scores was statistically significant (*p* < 0.0001). The CsA group tended to improve corneal opacity, but without significant difference (Figure 5A,B). A MILLIPLEX bead-based assay of the cytokines of rats showed that the scopolamine and Dendrobium treatment did not affect the expression of the cytokines in serum. However, the inflammatory IL-1β and TNF-α expression in the DLW-low group were significantly increased compared with the model group (*p* < 0.05) (Figure 5C).

HE staining of paraffin sections is one of the common clinical–pathological detection methods, mainly used to detect the morphological changes in the lacrimal gland, retina and conjunctival epithelial cells (Figure 5D–F). In the present study, we observed no acinar atrophy or acinar fibrosis in the lacrimal gland of the control group, no ductal or periacinar fibrosis and no polymorphonuclear cells or lymphocytes infiltration around the nucleus and blood vessels. In the model group, the size and shape of the acinar and ductal cells were irregular. Nuclei and perivascular infiltration of the polymorphonuclear cells and lymphocytes were observed. The volume of the cells was obviously shrunken, the cells were clustered, the borders of some cells were unclear and the morphology of the nuclei was abnormal. All treatment groups could improve the morphological changes of the lacrimal gland in the model group. As shown in Figure 5E, the retinas of the rats in the control group have a good layer, about 4 or 5 layers, neatly arranged and clearly layered, and the cells at the base are in normal shape and neatly arranged in a columnar shape. The conjunctival epithelial cells were neatly arranged, and the goblet cells were intact, distributed among the epithelial cells. Compared with the control group, the model group showed morphological changes in the outer nuclear layer of the retina, some cells in the basal layer were missing, and vacuoles appeared. The conjunctival epithelium showed hyperplasia, increased cell thickness, disordered layers, disordered arrangement of basal cells, damage and shedding of superficial cells, increased number of layers of superficial squamous cells, increased keratinization of conjunctival tissue and blurred layers of conjunctival epithelial cells (Figure 5F). Compared with the model group, the retinal structure of the rats in the DOW and DLW group was improved, the arrangement was relatively neat, and the pathological conditions were significantly improved, which was close to the normal level. DOW and DLW have a certain protective effect on the retinal and conjunctival epithelial cells of rats with dry eye. In the CsA group, there were still vacuolar changes in the outer nuclear layer of the retina, and conjunctival hyperplasia was obvious.

### 2.6. DOW and DLW Decrease MMP-9 and MMP-2 Expression and Modulate AQPs Expression in the Eyeball and Lacrimal Gland of SCOP-Induced DED Rats

**Figure 6:**
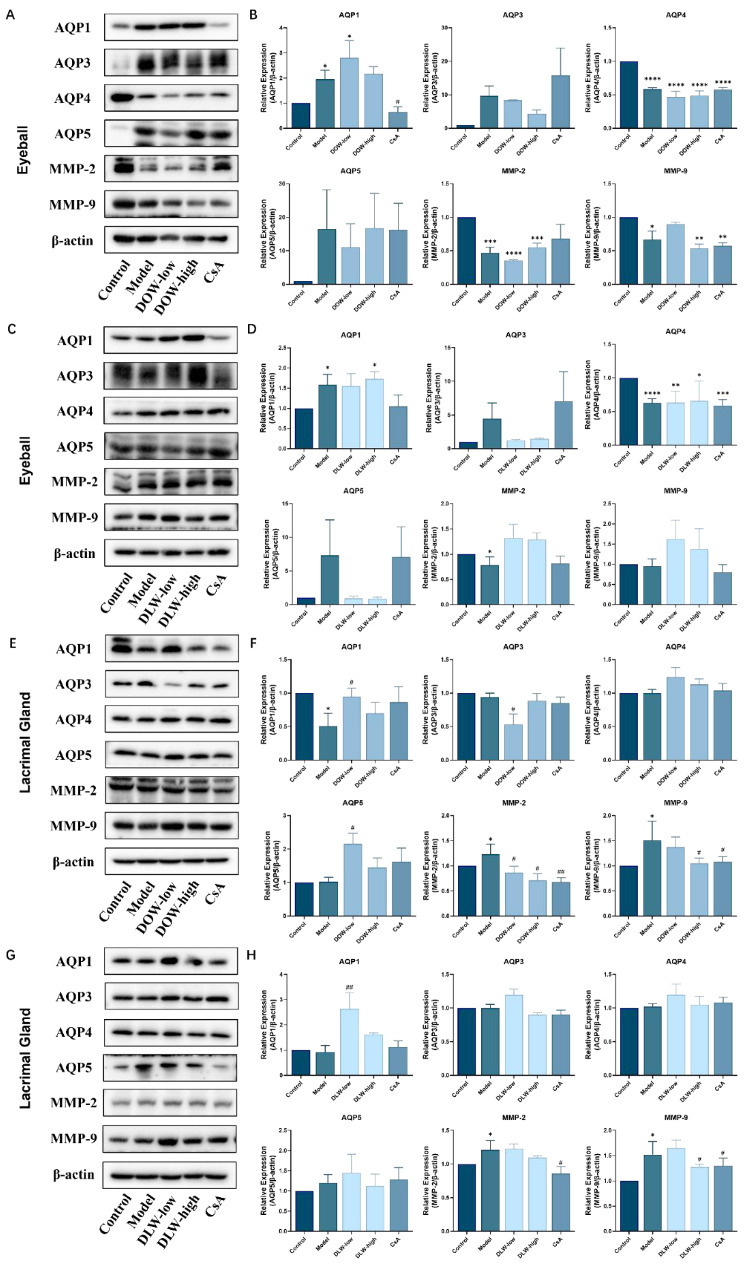
DOW and DLW decrease MMP-9 and MMP-2 expression and modulate AQPs expression in the eyeball and lacrimal gland of SCOP-induced DED rats. (**A**) The bands selected represent the effect of DOW on protein expression of AQPs and MPPs in eyeball by Western blot in each group (*n* = 3). (**B**) Quantitative statistics of protein expression in Figure 6A. The grey value was obtained by Image J software processing. Data were expressed as mean ± SEM from five rats in each group. * *p* < 0.05, ** *p* < 0.01, *** *p* < 0.001 and **** *p* < 0.0001 compared to control group individually; # *p* < 0.05, ## *p* < 0.01 compared to model group individually. (**C**) The bands selected represent the effect of DLW on the protein expression of AQPs and MPPs in eyeball by Western blot in each group (*n* = 3). (**D**) Quantitative statistics of protein expression in C. The grey value was obtained by Image J software processing. Data were expressed as mean ± SEM from five rats in each group. * *p* < 0.05, ** *p* < 0.01, *** *p* < 0.001 and **** *p* < 0.0001 compared to control group individually; # *p* < 0.05 compared to model group individually. (**E**) The bands selected represent the effect of DOW on the protein expression of AQPs and MPPs in lacrimal gland by Western blot in each group (*n* = 3). (**F**) Quantitative statistics of protein expression in Figure 6E. The grey value was obtained by Image J software processing. Data were expressed as mean ± SEM from five rats in each group. * *p* < 0.05, ** *p* < 0.01, *** *p* < 0.001 and **** *p* < 0.0001 compared to control group individually; # *p* < 0.05 compared to model group individually. (**G**) The bands selected represent the effect of DLW on protein expression of AQPs and MPPs in lacrimal gland by Western blot in each group (*n* = 3). (**H**) Quantitative statistics of protein expression in Figure 6G. The grey value was obtained by Image J software processing. Data were expressed as mean ± SEM from five rats in each group. * *p* < 0.05, ** *p* < 0.01, *** *p* < 0.001 and **** *p* < 0.0001 compared to control group individually; # *p* < 0.05 compared to model group individually.

The results in Figure 6A–D show that the protein expression of AQP4 in the eyeball of the model group was significantly decreased, compared with that of the control group (*p* < 0.05), and the DOW-low and DLW-high group could significantly upregulate the protein expression of AQP1 (*p* < 0.05). In the lacrimal gland (Figure 6E–H), the DOW-low and DLW-low group was able to significantly upregulate the protein expression of AQP1 compared with the model group (*p* < 0.05). There is also a significant decrease in AQP4 in lacrimal gland, and all the treatment could not reverse this situation. The DOW-low (*p* < 0.05) and DLW-low group was able to increase the protein expression of AQP5 compared with the control group. Furthermore, in lacrimal gland, the expression of MMP-9 and MMP-2 in the model group was significantly increased (*p* < 0.05). The DOW and DLW group could significantly decrease the expression of MMP-9 and MMP-2 protein (*p* < 0.05). In the eyeball, the expression of MMP-9 and MMP-2 protein in the model group decreased. While DOW could further reduce the protein expression of MMP-9 and MMP-2 (*p* < 0.01), DLW did not exhibit a similar effect.

### 2.7. DOW and DLW Inhibit MAPKs and NF-κB Signal Transduction in the Eyeball and Lacrimal Gland of SCOP-Induced DED Rats

**Figure 7:**
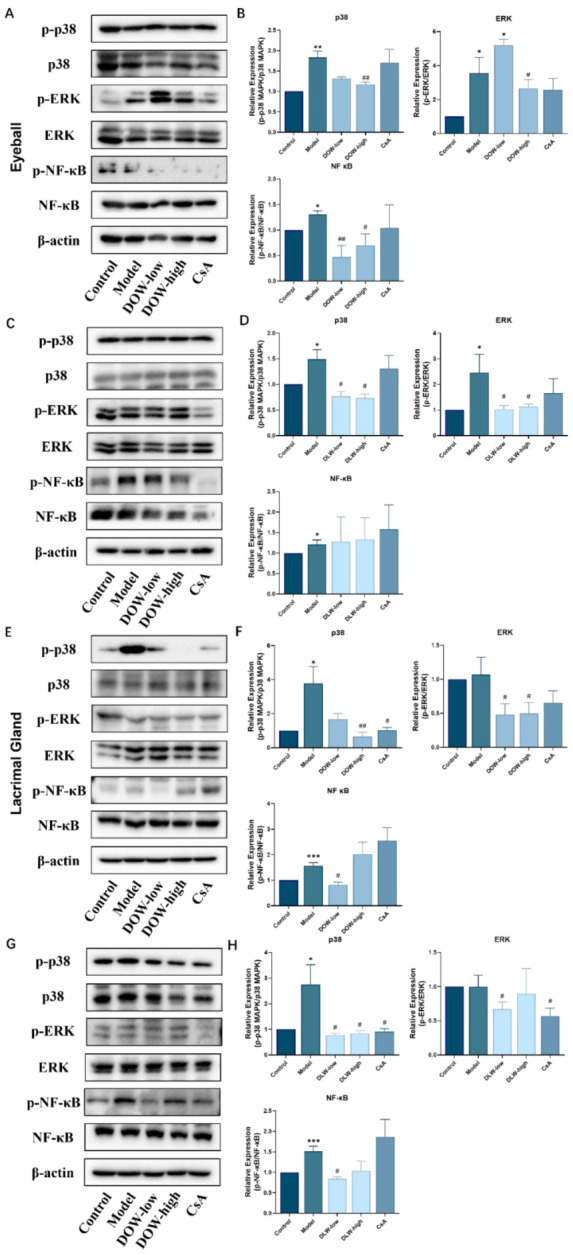
DOW and DLW inhibit MAPKs and NF-κB signal transduction in the eyeball and lacrimal gland of SCOP-induced DED rats. (**A**) The bands selected represent the effect of DOW on protein expression of MAPKs and NF-κB pathway in eyeball by Western blot in each group (*n* = 3). (**B**) Quantitative statistics of protein expression in Figure 7A. The grey value was obtained by Image J software processing. Data were expressed as mean ± SEM from five rats in each group. * *p* < 0.05 and ** *p* < 0.01 compared to control group individually; # *p* < 0.05 and ## *p* < 0.01 compared to model group individually. (**C**) The bands selected represent the effect of DLW on protein expression of MAPKs and NF-κB pathway in eyeball by Western blot in each group (*n* = 3). (**D**) Quantitative statistics of protein expression in Figure 7C. The grey value was obtained by Image J software processing. Data were expressed as mean ± SEM from five rats in each group. * *p* < 0.05 compared to control group individually; # *p* < 0.05 compared to model group individually. (**E**) The bands selected represent the effect of DOW on the protein expression of MAPKs and NF-κB pathway in lacrimal gland by Western blot in each group (*n* = 3). (**F**) Quantitative statistics of protein expression in Figure 7E. The grey value was obtained by Image J software processing. Data were expressed as mean ± SEM from five rats in each group. * *p* < 0.05 and *** *p* < 0.001 compared to control group individually; # *p* < 0.05 and ## *p* < 0.01 compared to model group individually. (**G**) The bands selected represent the effect of DLW on protein expression of MAPKs and NF-κB pathway in lacrimal gland by Western blot in each group (*n* = 3). (**H**) Quantitative statistics of protein expression in G. The grey value was obtained by Image J software processing. Data were expressed as mean ± SEM from five rats in each group. * *p* < 0.05 and *** *p* < 0.001 compared to control group individually; # *p* < 0.05 compared to model group individually.

The results in Figure 7A–D show that in the eyeball, compared with the control group, the model group could increase the expression of p-ERK, p-p38 and p-NF-κB (*p* < 0.05), and the DOW and DLW group could downregulate the activation of p-ERK, p-p38 and p-NF-κB (*p* < 0.05). In the lacrimal gland (Figure 7E–H), compared with the control group, the model group could increase the expression of p-p38 and p-NF-κB (*p* < 0.05). Compared with the model group, the DOW and DLW group could reduce the expression of p-ERK, p-p38 and p-NF-κB.

## 3. Discussion

In recent years, with changes in people’s living environments and lifestyles, the widespread use of video terminals and the aging of the population, the incidence of dry eye has been increasing rapidly. The causes of dry eye are complex, and most patients with moderate to severe dry eye have a prolonged disease, which is not satisfactory after long-term treatment and cannot be completely cured. In the 2014 consensus on the definition and diagnosis of dry eye developed by the Asian Dry Eye Society, dry eye was clearly defined as a chronic disease [2]. As an ophthalmic disease, dry eye affects patients’ quality of life and increases the economic burden of patients and affects socioeconomic development [24]. Therefore, the prevention and treatment of dry eye have become important topics among ophthalmologists.

Since the pathogenesis of dry eye has not been fully elucidated, it is crucial to establish an effective dry eye model to study its mechanism. Currently, there are several methods to establish animal models of dry eye [25,26,27], including tear gland removal, nerve blockade, parasympathetic drugs and induction of an autoimmune response. However, the hyperosmolarity of tears in human dry eye patients is currently considered the initial trigger that activates inflammatory mediators in corneal epithelial cells, increases various chemokines and triggers inflammation [28]. Therefore, in this study, to explore the protective effect of Dendrobium extracts on dry eye, we chose to establish an in vitro dry eye model by hyperosmotic solution induction, to ensure consistent modelling with the clinical situation. We focus on the regulation of aquaporins by Dendrobium extracts in vitro. Therefore, we selected human keratocytes (HKs), which can express AQPs, especially AQP1, AQP3, AQP4 and AQP5. Our in vitro study chose the sodium chloride concentration at 70% cell viability to induce the hyperosmotic cell model.

In our in vitro dry eye model, we found that the intracellular NF-κB and MAPKs signalling pathways were activated (Figure 2E). Our study found that DLW could inhibit the activation of NF-κB and MAPKs signalling pathways by downregulating the phosphorylation of their pathway-related cascade proteins, thereby exerting a protective effect against hyperosmolarity and reducing the protein expression of MMP-9. In our study, we found that the gene expression of AQPs was generally upregulated in an in vitro dry eye model, which would vary in different cells and might be related to the different distribution of AQP expression in different cells. AQPs mediate the passive transport of water across the cell membrane to maintain cellular homeostasis [29]. We found that the phosphorylation of ERK and the expression of AQP5 were significantly elevated in the hyperosmotic-condition-induced dry eye cell model, as well as the inflammatory IL-6, IL-8 and TNF-α, which promote the inflammatory response and apoptosis of cells. Therefore, ERK/AQP5 pathway plays an important role in the in vitro dry eye cell model. Upregulation of AQP5 by DLW exerts a unique function in promoting corneal wound healing by promoting cell migration and proliferation in the corneal fibroblast. High AQP5 expression promotes corneal cell migration and proliferation toward the wound site, thereby promoting HKs migration and repair [30].

In this study, we used a subcutaneous scopolamine injection for model induction, which is one of the current methods for immune–inflammatory modelling of dry eye. According to Qiu Jingjing et al. [31], the dry eye model made by this experimental method not only is caused by reduced tear secretion but also is accompanied by a combination of factors such as immune inflammation and apoptosis. Inflammatory cytokines cause the infiltration of cells in the ocular surface and the lacrimal gland and abnormalities in the nerve conduction function of the lacrimal gland, inducing dry eye and other pathological features. In addition, this method has the advantages of short modelling time, good stability and simple operation, so it has a wide range of applications.

In dry eye diseases, many mechanisms that maintain ocular surface and lacrimal gland homeostasis are disrupted. Both in animal experiments and clinical trials, it has been found that dryness of the ocular surface can cause a secondary immune response in the ocular surface and bacterial products, ultimately leading to a vicious cycle [32,33,34]. The hyperosmolarity of tears is a direct cause of the inflammatory response of the ocular surface epithelium, which leads to hyperpermeability of the corneal epithelium; activates signalling pathways, such as MAPKs and NF-κB; stimulates the release of inflammatory IL-1β, TNF-α and IL-6, chemokines, MMPs, such as MMP-2 and MMP-9; and causes apoptosis [32,33,34,35,36]. Our results showed that DOW and DLW were able to inhibit the expression of MMP-9 significantly and had an inhibitory effect on the expression of MMP-2.

This experiment is the first study to evaluate the efficacy of DOW and DLW on an animal model of SCOP-induced experimental dry eye. By observing the improvement of the ocular surface of rats with dry eyes by oral administration of DOW and DLW, a basis can be laid for optimizing the anti-inflammatory treatment of dry eyes and providing new anti-inflammatory drugs for dry eyes. Cyclosporine A is a new immunosuppressive agent that inhibits antibody formation and immune response [37]. It has been shown that the use of cyclosporine A effectively reduces the concentration of immune activation markers, apoptotic markers and inflammatory cytokines in ocular surface tissues, especially for the apoptosis of both conjunctival goblet cells and acinar cells of the lacrimal gland, suppressing ocular surface inflammation [38,39]. In addition, cyclosporin A (CsA) is approved by the FDA for clinical use in dry eye treatment. Therefore, CsA was selected as a positive control drug in this in vivo experiment.

Our results suggest that DOW and DLW exert an effect of increasing tear production, along with the effect of repairing the integrity of the corneal epithelium. The lacrimal gland plays a vital role in maintaining ocular surface health and protecting the eye from environmental exposure. It secretes proteins and fluids into the tear film, which is essential for maintaining a healthy ocular surface and a smooth refractive interface for light to enter the eye [40]. There are many studies on the relationship between diabetic dry eye and retinopathy [41,42]. Among them, the damage of lacrimal gland function directly causes insufficient tear secretion and changes the osmotic pressure of the ocular microenvironment. The hypertonic environment constantly stimulates the ocular surface, destroys the stability of the tear film, promotes the apoptosis of ocular cells and affects the normal function of other ocular structures. In addition, prolonged exposure to hypertonic environments can induce retinopathy. The modelling method used in our experiments was established by subcutaneous injection of SCOP. SCOP works directly on the lacrimal gland, inhibiting the normal function of the lacrimal gland and reducing the secretion of tears. During the dissection, we found that in the model group, the size of the lacrimal glands of the rats was significantly reduced. This led us to further investigate whether the structure of the rat eye could remain normal, even when the shape, size and function of the lacrimal gland were significantly inhibited. The model group showed morphological changes in the outer nuclear layer of the retina compared with the normal group, which indicated that the dysfunction of lacrimal gland may cause the structure changes in eyeball. The activation of inflammatory signalling pathways, such as p38 MAPK and NF-κB, is involved in the development of dry eye inflammation. Our experimental results showed that oral administration of DOW and DLW could improve the imbalance of the ocular surface microenvironment in dry eyes. It reduces the secretion of pro-inflammatory factor MMPs by inhibiting the activation of the NF-κB signalling pathway. Animal experiments revealed that the SCOP-induced dry eye model in rats was able to activate ocular surface-inflammation-related signalling pathways, such as extracellular signal-regulated kinase (ERK) and p38 MAPK. Moreover, the loss of conjunctival goblet cells in rats in the model group was also associated with inflammation-induced apoptosis. Therefore, DOW and DLW treatment may protect goblet cells and normal function by inhibiting ocular surface inflammatory pathways, such as ERK and p38 MAPK, thereby suppressing ocular surface inflammation. The results of NF-κB protein expression in ocular and lacrimal gland tissues showed that p-NF-κB/total NF-κB was elevated in the model group compared with the control group, indicating an increase in relative NF-κB activity. Moreover, DOW could reduce the relative expression of phosphorylated NF-κB, thereby indicating that DOW could inhibit the NF-κB signalling pathway.

In our investigation, we noticed that the effects of extracts on inflammatory response were not that obvious and direct. So, given that, our focus is mainly on the effect of the modulation of aquaporin expression and possible signal transduction. In a future study, we may study the signalling pathways that contribute to the initiation of inflammation, including the Toll-like receptor and interferon, as well as inflammasome activation [43]. We may further explore the specific roles that the RNA-binding protein PCAT plays in such proinflammatory signalling and in hyperosmotic stress-induced inflammation. Moreover, we may further investigate the other members in NF-κB signalling to examine if the Rel/NF-κB transcription factor family plays a regulatory role in dry-eye-related innate immunity, stress-induced intercellular signalling and transcriptional regulation [44]. In our study, to explore the protective effect of Dendrobium extracts on dry eye, we chose to establish an in vitro dry eye model by hyperosmotic solution induction to ensure consistent modelling with the clinical situation, in which the hyperosmolarity of tears is currently considered the initial trigger in dry eye patients. In the HK cell model, we focus on the protective effect of Dendrobium extracts and promotion of the cell migration. Therefore, we have not investigated Dendrobium’s effect on the osmoadaptive Slc38a2 mRNA level. However, the effect of the extracts on the Slc38a2 mRNA levels may also require further investigation. Dry eye disease is a multifactorial disease of the tears and ocular surface that causes tear-film instability, resulting from decreased lacrimal secretion or excessive lacrimal evaporation. In this animal model, this mechanism is more related to decreased secretion rather than evaporation. Therefore, we have not investigated the effect of Dendrobium extracts on lipid metabolism in the SCOP-induced rat model. The effect of extracts in the administrative form of eye drops on the stability of the tear film, lipid-metabolism-related fatty acid omega hydroxylase and biosynthesis of O-acyl-omega-hydroxy fatty acids should be investigated in the future. This study showed that administration of DOW and DLW inhibited inflammation-related signalling pathways in an animal model of experimental dry eye and in vitro hyperosmotic cell model, reducing the inflammatory response and, thus, effectively improving ocular surface damage. It has been reported that Dendrobium may contain polysaccharides and coumarin derivatives [45]. The pharmacological efficacy of herbs depends on the chemical composition, which is highly variable under the influence of genetic factors, harvesting time, culture conditions, origins, extraction methods and conditions, so quality control of herbs is crucial for better research into and application of traditional Chinese medicine. In this case, more effective qualitative and quantitative methods should be developed to evaluate the quality of Dendrobium species. This study provides a new perspective on dry eye treatment, and DOW and DLW may be developed as a potential therapeutic agent for dry eye.

## 4. Materials and Methods

### 4.1. Herbs and Reagents

The original herbs of Dendrobium officinale Kimura et Migo and Dendrobium loddigesii Rolfe were purchased from Guangzhou Zhixin Chinese Herbal Beverage Company. Scopolamine hydrobromide (SCOP), emodin and cyclosporin A (CsA) were all purchased from Sigma-Aldrich (St. Louis, MO, USA).

### 4.2. Preparation of Dendrobium Extracts

Dendrobium officinale Kimura et Migo water extract (DOW) and Dendrobium loddigesii Rolfe water extract were used in this experiment as a therapeutic agent for cells and Sprague Dawley (SD) rats. Dried Dendrobium officinale Kimura et Migo and Dendrobium loddigesii Rolfe (1 kg) were cut into pieces, the water extract was extracted twice with distilled water (1:10) and each extraction was boiled for 2 h. The extracts were combined, filtered and further centrifuged at 1500 rpm to remove the insoluble powder. The water extracts were lyophilized into powder and stored in sealed bags at −20 °C.

### 4.3. Experimental Animals

The experimental animals used in this study were healthy 6-week-old male SD rats, weighing 160–180 g, from the Animal Center of the Chinese University of Hong Kong and housed in the Animal Center of the Institute of Chinese Medicine at the Chinese University of Hong Kong. The rats were examined before the experiment and were free of eye diseases, including eye inflammation and other abnormalities. All experimental animal procedures were carried out according to the Animal Protection and Use Committee regulations. All animal experiments were conducted in accordance with the principles outlined in the Animal Experimentation Ethics Committee Guide for the Care and Use of Laboratory Animals, as approved by the Animal Experimentation Ethics Committee of the Chinese University of Hong Kong.

### 4.4. Establishment of Dry Eye Animal Models and Grouping Protocol

The experimental dry eye model of rats with subcutaneous scopolamine injection was applied, as previously reported [46]. All rats were divided into seven groups: standard control group, dry eye model group (model), DOW-low group, DOW-high group, DLW-low group, DLW-high group and cyclosporine A (CsA) group (*n* = 5 per group). During the experiment, all rats, except the standard control rats, were injected subcutaneously with scopolamine (concentration: 8.4 mg/mL; 3 times a day: 11 am, 2 pm and 5 pm; 0.5 mL each time) for five days to induce a short-term dry eye animal model. Moreover, for rats in the DOW, DLW and CsA groups, treatment was started on the third day of the experiment. The DOW/DLW group was orally administrated with a dose of DOW (200 mg/kg/day for high dose and one-third of high dose for low-dose treatment), once a day at 3 pm. The CsA group was treated with daily eye drops (0.05%), administered twice daily at 11 and 4 pm. The duration of treatment was from the 3rd day of the experiment to the 14th day of the experiment, lasting for 12 days. All rats were placed under normal environmental conditions (25 ± 2 °C, 50 ± 5% relative humidity, 12 h of light circadian cycle, good ventilation).

### 4.5. Experimental Cells

Human cornea keratocytes (HKs), was purchased from ScienCell (USA). The cells were cultured in complete medium consisting of basal medium (DMEM), 10% fetal bovine serum (FBS), 1% penicillin (100,100 U/mL) and 1% streptomycin (100 μg/mL), with medium changes every other day.

### 4.6. MTT Assay for the Detection of Drug Toxicity of Dendrobium Extracts

MTT assay was used to detect the drug toxicity of Dendrobium extract on cells, in order to optimize the concentration of Dendrobium extract adopted for in vitro experiments. The cells were inoculated into 96-well plates, and each well was treated with different concentrations of Dendrobium extract for 24 h or 48 h. Then, MTT reagent (20 μL) was added, and the cells were incubated for 4 h in the incubator. The absorbance of OD 540 nm was measured, and the cell survival rate of each group was calculated.

### 4.7. Preparation of Hyperosmolarity-Induced Dry Eye Cell Models (Selection of Optimal Hyperosmotic Concentration)

The human cornea keratocytes were inoculated into 96-well plates and were incubated in the cell culture incubator for 12–24 h. The old medium was discarded, the cells were treated with medium prepared with different concentrations of sodium chloride (NaCl) and the control group was incubated with the medium and continued to be incubated in the cell culture incubator for 24 h and 48 h, respectively. Then, MTT reagent (20 μL) was added and incubated in the cell culture incubator for 4 h. The absorbance of OD 540 nm was measured, and the cell survival rate of each group was calculated.

### 4.8. Cell Scratch Assay

Cells were inoculated in 6-well plates and incubated overnight without serum starvation at 90%–100% fusion. After 12 h, a quick and forceful uniform scratch was made with a 200 μL sterile pipette tip perpendicular to the plate. Wash 2 to 3 times with PBS to wash away the suspended cells. Pictures at 0 h were taken by optical microscopy. After that, the cells were treated with different concentrations of Dendrobium extracts accordingly for 24 h. After reaching the time point, the medium was aspirated and photographed by optical microscopy. For image processing, the area of the scratch channel was circled by the software for each photo of the same location at different moments, the area of migration = area of 0 h–area of 24 h and the final result was expressed as migration area/area of 0 h scratch channel for migration rate.

### 4.9. Real-Time Polymerase Chain Reaction (RT-PCR)

After the cells were treated with different concentrations of Dendrobium extracts for 24 h, the total RNA of cells was extracted using an RNA extraction kit (Takara Bio Inc., San Jose, CA, USA). RNA concentration was detected, and subsequent experiments were performed according to ExScript Reagent Kit (Perfect Real Times, Takara). The primers of AQP1, AQP3, AQP4, AQP5 and GAPDH were ordered from BGI, Shenzhen, China. The specific sequences are shown in Table 1. The reaction was performed according to the following condition: 95 °C pre-denaturation for 10 s; cycling conditions: 95 °C for 5 s, 60 °C for 35 s with simultaneous fluorescence signal detection and 40 cycles of reaction. The dissociation curve and amplification curve were plotted at the end of the reaction. The CT value represents the number of cycles when the fluorescence signal reached the set domain value in each reaction tube. Three replicate wells were made for each sample, and the average CT value was taken and brought into the calculation to obtain the ploidy of gene expression in the treated group compared with the standard control group.

### 4.10. Western Blot Analysis for Protein Detection

The amount of protein extraction solution (RIPA) required for the experiment was taken out in advance for precooling, and suitable concentrations of protease inhibitors and phosphatase inhibitors were added. After the cells were treated with different concentrations of Dendrobium extracts for 24 h, the cells were collected, and working solution (100 μL) was added to each cell sample. Half of the lacrimal gland and the whole eye were used for protein extraction, and working solution (400 μL) was added to each homogenizer to homogenize centrally until each homogenizer was free of tissue clumps. The cells and tissues were centrifuged at 4 °C for 5 min at 14,000× *g*. The supernatant was collected and protein concentration was determined by the BCA method. A 50 μg protein sample was subjected to a running gel phase with 10% isolate gel and 5% concentrate gel, followed by transferring the protein to a PVDF membrane, after which the membrane was blocked with 5% skim milk for one hour. The membrane was cut open according to the desired protein molecular weight. The information of primary antibody (1:1000) and secondary antibody (1:2000) are as follows: AQP1 Rabbit mAb (A4195), AQP3 Rabbit pAb (A2838), AQP4 Rabbit pAb (A2887), AQP5 Rabbit pAb (A9927), ERK1 / ERK2 Rabbit pAb (A16686), p38 MAPK Rabbit pAb (A14401), phosphor-ERK1-T202/Y204 and ERK2-T185/Y187 Rabbit mAb (AP0974), phosphor-p38 MAPK-T180/Y182 Rabbit pAb (AP0526), NF-kB p65/RelA Rabbit mAb (A19653), phosphor-NF-kB p65/RelA-S276 Rabbit pAb (AP0123), MMP2 Rabbit pAb (A6247), MMP9 Rabbit pAb (A0289), and HRP Goat Anti-Rabbit IgG (H + L) (AS014) were all purchased from Abclonal Technology Co., Ltd. (Woburn, MA, USA).

### 4.11. Detection of Tear Secretion

The tear secretion of each group of rats was measured by the tear-detection paper-strip method. The tear-detection paper strip (Tianjin Jingming New Technology Development Co., Ltd., Jin Food and Drug Administration Machinery Production License No. 20100040) was designed according to Schirmer’s I experiment. The rat was fixed in the unanesthetized state, and then the tear test strip was placed into the rat’s eyelid at one-third of the reach, allowing the eyelid to close naturally. The time was recorded for 30 s, the strip was removed and placed on a ruler to measure the part of the moist filter paper strip, and the data was recorded. Tear secretion was measured throughout the experiment on days 1, 3, 7 and 15.

### 4.12. Measurement and Scoring of Corneal Opacity

The rats were anaesthetized with a ketamine–xylazine combination, and then the corneal opacity was observed using a slit lamp under a white light filter. The photographs taken were subsequently scored on the following criteria: (1) iris details were visible; (2) iris details were slightly blurred, and translucent areas of the eye were discernible; (3) iris details were not obvious, and pupil size was barely visible; and (4) iris was not visible and opaque [47].

### 4.13. Corneal Epithelial Fluorescein Staining Score

After 20 μL of 1% sodium fluorescein saline solution was dropped into the conjunctival of rats for 90 s, corneal epithelial sodium fluorescein staining was observed under the cobalt blue filter of a slit lamp microscope and photographed. Afterwards, the photographs were scored, the corneas were divided into four quadrants and scored separately, and the final score was obtained by the summation of all scores. Scoring method: each quadrant could accumulate 0–4 points, for a total of 0–16 points in the four quadrants. Rules for scoring: 0 points for no staining; 1 point for ≤30 punctate staining; 2 points for >30 punctate staining; 3 points for forming very diffuse staining but excluding the presence of stained plaques; and 4 points for forming stained plaques [48].

### 4.14. Hematoxylin–Eosin (HE) Staining

Paraffin sections to be stained were washed twice in xylene for 10 min, then washed in 100%, 95%, 90%, 80% and 70% ethanol for 3 min, and washed in tap water for 10 min. Hematoxylin staining for 5 min and washing in tap water for 1 min were performed. Alcohol fractionation with 1% hydrochloric acid for 30 s, washing with tap water for 1 min, PBS returned to blue colour for 30 s and washing with tap water for 1 min were performed. Eosin staining for 2 min and tap-water washing for 3 min were performed. Alcohol dehydration and xylene treatment of sections, to make them transparent, were performed. The pathological histological changes of the rat eye and lacrimal gland were observed under the microscope.

### 4.15. Periodic Acid-Schiff (PAS) Staining

The paraffin sections to be stained were washed twice in xylene for 10 min, then in 100%, 95%, 90%, 80% and 70% ethanol for 3 min and in tap water for 10 min. Periodate was used for treatment for 4 min and repeated three times. Schiff’s stain was added for staining for 15 min and repeated 3 times. Mayer’s hematoxylin was added dropwise for 5 min and washed with tap water for 2 min. Dehydration in ethanol and treatment of sections with xylene were used to make them transparent. Three sections were taken under a microscope, and each section was counted in three high magnification fields. The average value was taken as the representative value of the number of conjunctival goblet cells.

### 4.16. Immunohistochemical Staining

Paraffin sections were routinely dewaxed, as described previously [49], placed in sodium citrate buffer, heated in microwave oven for 20 min for antigen repair and left to cool at room temperature. Then, they were incubated in 3% hydrogen peroxide deionized water for 10 min to eliminate endogenous peroxidase activity and rinsed with PBS for 3 min for 3 times. Standard goat serum working solution was added for blocking by incubation for 15 min at room temperature. Rabbit monoclonal primary antibody was added, and muc5ac (1:200, antibody from Thermo, USA) was diluted in the appropriate ratio and incubated overnight at 4 °C. The next day, PBS was used to rinse it for 3 times. HRP-enzyme-labeled goat anti-rabbit IgG was added, incubated for 30 min at 37 °C and rinsed with PBS for 3 times. Diaminobenzidine (DAB) was developed and rinsed well with tap water. Hematoxylin restaining, gradient alcohol dehydration, xylene transparency and neutral gum sealing were performed. The positive results were obtained by dark brown staining of the cytoplasm, and the expression of muc5ac in each lacrimal gland and eye tissue group was observed under the microscope.

### 4.17. Statistical Analysis Method

The data results of each group were counted, and GraphPad Prism 9 was used for statistical analysis of data to compare whether the data differences between groups were statistically significant. Quantitative data were expressed in the form of mean ± standard deviation, and ANOVA was used. Student’s *t*-test was used to compare between groups, and *p* < 0.05 was considered statistically significant.

## 5. Conclusions

Dry eye is a chronic inflammatory disease with a high recurrence rate and requires long-term treatment. In conclusion, this study showed that oral administration of DOW and DLW can enhance tear production in rats and has a protective effect on ocular surface damage. DOW and DLW can enhance the expression of AQP5 and muc5ac proteins in the lacrimal gland and reduce the inflammatory response. At the same time, DOW and DLW can protect the goblet cells in the conjunctiva. DOW and DLW can inhibit the activation of the corresponding intracellular MAPKs pathway, inhibit the inflammatory NF-κB pathway and play a role in improving dry eye symptoms.

## Figures and Tables

**Table 1:** Primer sequences for RT-PCR.

Name		Sequence (5′ to 3′)
GAPDH	Forward	TGATGACATCAAGAAGGTGGTGAAG
	Reverse	TCCTTGGAGGCCATGTAGGCCAT
AQP1	Forward	TGCCATCGGCCTCTCTGTAG
	Reverse	AAGGACCGAGCAGGGTTAATC
AQP3	Forward	GGGGAGATGCTCCACATCC
	Reverse	AAAGGCCAGGTTGATGGTGAG
AQP4	Forward	AGCAGTCACAGCGGAATTTCT
	Reverse	TCTGTTCCACCCCAGTTGATG
AQP5	Forward	CGGGCTTTCTTCTACGTGG
	Reverse	GCTGGAAGGTCAGAATCAGCTC

## Data Availability

The data presented in this study are available on request from the corresponding author.
